# Variants of CDKAL1 rs7754840 (G/C) and CDKN2A/2B rs10811661 (C/T) with gestational diabetes: insignificant association

**DOI:** 10.1186/s13104-018-3288-7

**Published:** 2018-03-15

**Authors:** Amr El Noury, Osama Azmy, Jehan Alsharnoubi, Sameh Salama, Ahmed Okasha, Weaam Gouda

**Affiliations:** 1 National Institute of Laser Enhanced Science, Cairo University, Cairo, Egypt; 2 Reproductive Health Department, National Research Centre, El Buhouth St., Dokki, Giza, 12622 Egypt; 3 Biochemistry Department, National Research Centre, El Buhouth St., Dokki, Giza, 12622 Egypt

**Keywords:** Allele frequency, Genetic association, CDKAL1, CDKN2A/B, Single nucleotide polymorphism (SNP), Gestational diabetes mellitus (GDM)

## Abstract

**Objectives:**

Pathophysiological similarity exists between gestational diabetes mellitus (GDM) and type 2 diabetes mellitus with common genetic origin. Genetic liability for GDM in our population is still not researched. The goal was to reveal the genotypic and allele frequency differences of 2 single nucleotide polymorphisms (SNPs) namely, CDKAL1 (rs7754840) and CDKN2A/2B (rs10811661) between GDM pregnancies and normal pregnancies. We assessed them by real time polymerase chain reaction using Taqman^®^ allelic discrimination assays. We included 47 GDM pregnant subjects and 51 normal glucose tolerance (NGT) pregnant women as controls.

**Results:**

The genotype frequencies in the GDM group and the NGT group of rs7754840-GG/GC/CC were 6.4/15.7% (3/8), 55.3/45.1% (26/23) and 38.3/39.2% (18/20) respectively. Also, those of rs10811661-CC/CT/TT were 74.5/14.9/4.3% (38/7/2) and 80.9/19.6/5.9% (38/10/3) respectively. The allele frequencies in the GDM group and the NGT group of C/G and T/C were 66/34% (62/32), 61.8/38.2% (63/39) and 11.7/88.3% (11/83), 15.7/84.3% (16/86) respectively. There were no statistical differences between the two groups in allele frequencies and genotype frequencies (all P > 0.05). Non-significant association was seen in the two SNPs of *CDKAL1* and *CDKN2A/B* genes with GDM. Further studies are essential to validate data.

## Introduction

A well known subtype of diabetes mellitus (DM) is gestational diabetes mellitus (GDM) that could result in serious morbidity for the mother and fetus [1]. Obesity increases the incidence of the condition with a strong association of disease occurrence [2, 3]. The pathophysiologic pathway of developing GDM is still not well understood. The condition may result in serious impact on pregnant ladies and their offsprings, e.g. high blood pressure, cesarean delivery, preterm labor, macrosomia and hyperbilirubinemia, in addition to the liability to develop metabolic syndrome and type 2 diabetes [1, 4]. GDM is a cornerstone risk factor of various obstetric diseases like polyhydramnios, neonatal hypoglycemia, hypocalcaemia, raised red blood cell count, intellectual disability, birth trauma and increased mortality rates [5].

DM subtypes GDM and type 2 diabetes mellitus (T2DM) share common background of risk factors like diabetic family history, abnormal glucose tolerance, increased BMI, and specific races having higher predilection. The pathophysiology is commonly similar between GDM and T2DM, this may indicate further genetic variability in GDM which may also act as a predictor for T2DM [6].

Lately genome-wide association studies (GWAS) and large-scale genetic linkage analyses regarding GDM have not been sufficiently explored. A targeted gene approach of identified genes of T2DM has approached the majority of GDM genetic causes [7].

So, GDM has also a genetic background. This was clear in studies showing grouping of T2DM in families with GDM. Mothers of women with GDM were also found to have higher prevalence of T2DM [8].

GWASs shows that the risk allele of rs7754840 of cdkal1 is associated with reduction in insulin secretion [9], as well as decrease in first-phase insulin release by 24%, higher glucose area under the curve and impaired insulin release [10]. There is association of T allele of the rs10811661 to T2DM [11]. It has also a role in pancreatic cancer [12].

In this study, we compared the single nucleotide polymorphisms (SNPs) in the diabetic genes rs7754840 in CDKAL1 [cyclin-dependent kinase 5 regulatory subunit associated protein-1-like1] and rs10811661 in CDKN2A/2B [cyclin-dependent kinase inhibitor 2A and 2B] in GDM patients with those in normal pregnant women as controls.

## Main text

### Methods

#### Study subjects

In their second trimester of 24–28 gestational weeks and age group between 18 and 45 years, a total of 47 women with GDM were selected. Fifty-one pregnant women with normal glucose tolerance (NGT) were taken as controls to account for the environmental influence (i.e. pregnancy). All cases were recruited over a period of 12 months from The Medical Research Center of Excellence of The National Research Center and Kasr Al-Eini Hospital, Cairo University. Screening of diabetes during pregnancy was done according to the American Diabetes Association guidelines [13]. Women with type 1 diabetes mellitus were excluded from the study.

#### Demographic data

Clinical data of all subjects, age, height, weight at 1 year before pregnancy, systolic blood pressure and diastolic blood pressure, were recorded. Also, gravidity and parity, family history of diabetes and history of gestational diabetes in each subject were recorded. Complete fetal anomaly scan by ultrasound was done.

*Blood samples* Blood samples of 2 mL were collected from the pregnant women in ethylene diamine tetra acetic acid (EDTA) tubes.

#### Isolation of genomic DNA

Genomic DNA was isolated from peripheral white blood cells using QIA amplification extraction kit (QIAamp^**®**^ DNA Blood Mini Kit (50) Cat. No. 51104) according to the manufacturer’s instructions and the isolated DNA was subsequently quantified using NanoDrop™ 2000 (Thermo Scientific, Wilmington, Delaware, USA).

#### Genotyping

*DNA analysis for CDKAL1 (rs7754840) and CDKN2A/2B (rs10811661)* Screening of rs7754840 and rs10811661 was performed with the TaqMan^**®**^ allelic discrimination assay (TaqMan^**®**^ universal Master Mix, no UNG, Part No.: 4440043, Applied Biosystems, Foster City, CA). The genotyping reaction was amplified on a GeneAmp PCR system 2700 (95 ^°^C for 10 min, followed by 40 cycles at 95 ^°^C for 15 s and 60 ^°^C for 1 min). Fluorescence was detected on an ABI Prism 7000 sequence detector (Applied Biosystems, USA). The genotyping success rate was 99.7 and 100% in the two studied groups respectively, and the error rate was 0% in both studied groups.

#### Statistical analysis

All statistical analyses were performed by SPSS 20.0 for Windows. The statistical power and sample size were calculated using PASS 11. The Chi square test was used for the comparison of expected and observed frequencies of categorical variables. A value of p < 0.05 (two-tailed) was considered statistically significant. A multiple logistic regression model was used to investigate the individual effect of these genes on GDM. These analyses were based on additive, recessive and dominant models, and adjusted for age and the family history of type 2 diabetes. The ORs with 95% confidence intervals (CIs) were presented.

### Results

The data of participants are summarized in Table 1. The cases and controls were well matched in age, body mass index (BMI), gestational week and gravidity. There was a significant difference between pre-BMI women in the GDM group compared to controls (non-GDM) (p < 0.01), whereas obesity was similar in both groups (p = 0.141). Although, history of GDM in women with GDM group was less than NGT group with significant change (< 0.0001).

**Table 1:** Characteristics of the study groups

	Group		P value	OR	95% CI
	GDM (%)	NGT			
Age group^a^ (years)					
18–20	2 (4.3%)	6 (11.8%)	0.019	–	–
21–30	20 (42.6%)	32 (62.7%)			
31–40	22 (46.8%)	13 (25.5%)			
>40	3 (6.4%)	0 (0%)			
Pre-BMI^a^ categories					
Underweight ≤ 18.5	0 (0.0%)	2 (3.9%)	0.01	–	–
Normal weight = 18.5–24.9	10 (21.3%)	21 (41.2%)			
Overweight = 25–29.9	36 (76.6.0%)	23 (45.1%)			
Obesity = BMI of 30 or greater	1 (2.1%)	5 (9.8%)			
Obesity					
Obese	34 (72.3%)	29 (56.9%)	0.141	1.9	0.852–4.623
None obese	13 (27.7%)	22 (43.1%)			
Family History of DM					
Yes	20 (42.6%)	16 (31.4%)	0.297	1.92	0.709–3.705
No	27 (57.4%)	35 (68.6%)			
History of GDM					
Yes	18 (38.3%)	0 (0%)	0.000	2.789	2.063–3.689
No	29 (61.7%)	51 (100%)			

^a^Risk estimate statistics cannot be computed. They are only computed for a 2 * 2 table without empty cells

#### Allele and genotypic association of different SNPs

The allele and genotype frequencies of the two SNPs are shown in Table 2. The frequencies of the heterozygote (GC) of the two SNPs of *CDKAL1* gene were found to be nonsignificantly higher in patients than in the controls (non-GDM) (*P* **=** *0.184*), suggesting risk manipulating nature. Although a similar pattern of combination was not observed in the homozygote genotype frequency (TT) of *CDKN2A/B* gene, the heterozygote (CT) was found to have the least frequency.

**Table 2:** Comparison of genotype frequencies between GDM cases and NGT group

Gene/allele	SNP	Genotype	GDM	NGT	P	P value	OR (95% CI)
CDKAL1 Genotype	rs7754840	GG/GC/CC	3/26/18 (6.4/55.3/38.3)	8/23/20 (15.7/45.1/39.2)		0.301	
		GG	3 (6.4)	8 (15.7)	0.132		
		GC	26 (55.3)	23 (45.1)	0.184		
		CC	18 (38.3)	20 (39.2)	0.746		
Allele		C	62 (66)	63 (61.8)		0.542	1.199 (0.669–2.152)
		G	32 (34)	39 ( 38.2)			
CDKN2A/B Genotype	rs1081166	CC/CT/TT	38/7/2 (74.5/14.9/4.3)	38/10/3 (80.9/19.6/5.9)		0.753	
		CC	38 (74.5)	38 (80.9)	1.00		
		CT	7 (14.9)	10 (19.6)	0.467		
		TT	2 (4.3)	3 (5.9)	0.655		
Allele		T	11 (11.7)	16 (15.7)		0.419	0.712 (0.312–1.625)
		C	83 (88.3)	86 (84.3			

The presence of the risk alleles of *CDKAL1* SNPs, rs7754840 C allele and T allele of rs10811661 of *CDKN2A/B* SNP were relatively not higher in GDM patients than in the NGT group and the logistic regression analysis yielded non-significant odds ratios implying that the variant alleles did not show risk for developing GDM. The allele distributions in loci rs7754840 (CDKAL1) and rs10811661 (CDKN2A/B) displayed non-significant discrepancy between the GDM and NGT groups (P > 0.05). Even, alleles C&T were a common risk allele for both loci. The odds ratio (OR) values were 0.199 [95% confidence interval (CI): 0.669–2.152] for rs7754840 (CDKAL1) and 0.712 (95% CI: 0.312–1.625) for rs10811661 (CDKN2A/B) (Table 2).

Table 3 summarizes variants association under additive, dominant recessive and over-dominant models. rs7754840 CDKAL1 SNPs were not significantly associated with GDM under the four models. In addition, CDKN2A/2B rs10811661 were not associated with GDM, but illustrated lower magnitude of effect under the additive model.

**Table 3:** Logistic regression of distributions and genetic models of each SNP’s genotype of *CDKAL1* and *CDKN2A/B*

		GDM, n (%)	None GDM, n (%)	P value	OR (95% CI)
*rs7754840 (CDKAL1)*					
Co-dominant model	G/G	3 (6.4)	8 (15.7)		1
	C/G	26 (55.3)	23 (45.1)	0.122	3.014 (0.714–12.731)
	C/C	18 (38.3)	20 (39.2)	0.236	2.400 (0.551–10.457)
Dominant model	CG + CC	44 (93.6)	43 (84.3)	0.145	2.729 (0.678–10.976)
	GG	3 (6.4)	8 (15.7)		1
Recessive model	CC	18 (38.3)	20 (39.2)	0.926	0.962 (0.426–2.170)
	GG + CG	29 (61.7)	31 (60.8)		1
Over-dominant model	CG	26 (55.3)	23 (45.1)	0.312	1.507 (0.679–3.344)
	GG + CC	21 (44.7)	28 (54.9)		1
*rs10811661(CDKN2A/B)*					
Co-dominant model	CC	38 (80.9)	38 (74.5)		1
	CT	7 (14.9)	10 (19.6)	0.510	0.700 (0.241–2.031)
	TT	2 (4.3)	3 (5.9)	0.665	0.667 (0.105–4.218)
Dominant model	CT + TT	9 (19.1)	13 (25.5)	0.452	0.692 (0.265–1.811)
	CC	38 (80.9)	38 (74.5)		1
Recessive model	TT	2 (4.3)	3 (5.9)	0.715	0.711 (0.114–4.454)
	CC + CT	45 (95.7)	48 (94.1)		1
Over-dominant model	CT	7 (14.9)	10 (19.6)	0.538	0.718 (0.249–2.070)
	CC + TT	40 (85.1)	41 (80.4)		1

Using the logistic regression analysis of the genotypes, the heterozygote (GC) and homozygote (CC) genotypes of CDKAL1 SNP reveal non significant odds ratio (*p *= *0.122* and *p *= *0.236 respectively*). The observed association remained non-significant even after adjusting for covariates. Also, the genotype distribution of rs10811661 (CDKN2A/2B) dif-fered between the GDM and non-GDM groups but with no significant changes in co-dominant, dominant, recessive and over-dominant models, with P values of 0122, 0.236, 0.145, 0.926 and 0.312 respectively, and OR values of 3.014 (95% CI: 0.714–12.731), 2.400 (95% CI: 0.551–10.457), 2.729 (95% CI: 0.678–10.976), 0.962 (95% CI: 0.426–2.170) and 1.507 (95% CI: 0.679–3.344), respectively. The findings revealed that the genotype frequency of *CDKN2A/B* gene showed a negligible significance, it was not significant in the logistic regression analysis, with or without covariates (Table 3). The genotype distribution of rs10811661 (CDKN2A/2B) contrasted between the GDM and non-GDM groups but with no significant changes in co-dominant, dominant, recessive and over-dominant models, with P values of 0.510, 0.665, 0.452, 0.715 and 0.538 respectively, and OR values of 0.700 (95% CI: 0.241–2.031), 0.667 (95% CI: 0.105–4.218), 0.692 (95% CI: 0.265–1.811), 0.711 (95% CI: 0.114–4.454) and 0.718 (95% CI: 0.249–2.070), respectively (Table 3).

### Discussion

Genome wide association studies GWAS studies have revealed the association of a number of unique genes with T2DM [14–17]. The SNPs were finally replicated in different populations with strong association with T2DM and the meta-analysis performed for each of these genes also confirmed the risk for developing T2DM. Previous studies also showed significant associations of CDKAL1 with minute reduction in insulin response. Past studies exposed that there was a relation between SNPs in the *CDKAL1* gene and T2DM. Those typically hyper lined in GWA studies, were also seen in Malaysian patients with GDM [6, 14, 16]. Many studies among Asian populations tested the association between rs7754840 in CDKAL1 and GDM risk [7, 18, 19]. There was a significant association between the risk of GDM and the C allele of rs7754840 with collective OR 1.40 (95% CI: 1.13–1.72), P value < 0.002 [20].

The decisive contrast over these studies appeared from the variability within the study populations; two of them were in Korean ladies with robust associations between GDM risk and rs7754840 [18, 19]. However, a Chinese study did not find any association [7]. This was in agreement with our results in the present study where we did not find any relation between rs7754840 and GDM.

Nevertheless, we did not detect a significant difference in these genotype and allele frequencies between GDM and non-GDM groups in Egyptian pregnant women (*P *> 0.05), indicating that the common susceptibility *loci* rs7754840 in *CDKAL1* may be not associated with GDM.

The main genetic variant significant with risk of T2DM {the loci CDKN2A/2B (rs10811661)} was examined in Caucasian populations [21, 22], Eastern Uttar Pradesh, India population and proved significant associated [23]. However, Nemr et al. [24]. demonstrated non significant association in the Lebanese population.

GWA studies described gene variants associated with T2DM containing *CDKAL1*, *CDKN2A/B* [9, 14–16, 25], yet, the association with GDM was not recognized. Variants with GDM including *CDKAL1* and *CDKN2A/B* genes in pregnant Malaysian women is not the same as GDM is dependent on ethnicity [18]. One of the primarily detected and widely analyzed deoxyribonucleic acid (DNA) markers is the common variant rs10811661 on the 9p21.3 locus. The marker is located in an intergenic region between the genes coding for the cyclin-dependent kinase inhibitors CDKN2A and CDKN2B [26]. CDKN2A/2B encodes two kinase inhibitors, which play an important role in β-cell regeneration [22], and thus the functional connection to type 2 diabetes is obvious.

This research is the first genetic study to approach GDM in Egypt. Our current findings highlighted the important contribution of some key genetic variants to GDM in Egyptian pregnant women. The findings of this present study provided preliminary insight into the GDM genetic variants in the Egyptian pregnant women. We did not find any risk of GDM associated with CDKAL1 (rs7754840) and *CDKN2A/2B* (rs10811661) (p value ≥ 0.0301–0.753), however, in previous GWAs studies those two SNPs were found to be related to T2DM [14, 16, 27]. In our study, the correlation was not established with GDM in pregnant women.

## Limitations

We observed that CDKAL1 (rs7754840) and CDKN2A/2B (rs10811661) gene polymorphism is unlikely to be associated with a risk of GDM in the pregnant women in our sample of Egyptian population. rs7754840 allele (C) and rs10811661 allele (T) were risk alleles for T2D in the pregnant women. Therefore, we assume that the lack of statistical significance is due to lack of power. We need to examine other GDM cohorts to confirm our results. Also, large scale studies should be done on pregnant women with T2DM.

The lack of reliable relation between rs7754840(C/G) and rs10811661(C/T) SNPs of CDKAL1and CDKN2A/2B genes may be explained by its dependence on other risk factors and genetic markers, which in fact determines the need for further investigations.

Finally, we tend to present this study to be a preliminary one, with a requirement for investigating a larger number of patients to confirm our results. Additional information on GDM-SNP association is required to optimize the SNP screening among pregnant ladies in Egypt. However, it is still uncertain whether or not this can be of value in developing countries where sure ethnicity of the population has much more risk to GDM.

## Abbreviations

BMI: body mass index
DM: diabetes mellitus
GWAS: genome-wide association studies
GDM: gestational diabetes mellitus
NGT: normal glucose tolerance
PCR: polymerase chain reaction
SNP: single nucleotide polymorphism
T2DM: type 2 diabetes mellitus
